# Transactions of the Chicago Gynæcological Society

**Published:** 1879-02

**Authors:** D. T. Nelson


### Article X.

### Transactions of the Chicago Gynæcological Society.

Third Meeting, December 27th, 1878. D. T. Nelson, m.d., in the chair.

Dr. E. O. F. Roler read a paper upon placenta prævia, with the report of a fatal case (published in the present number of the Journal and Examiner).

Dr. W. H. Byford: The subject of placenta prævia is one of great interest to every practitioner of midwifery, and our knowledge in reference to its management must be correct and immediately available to be of use to the patient. Dr. Roler's paper is commendable for its candor, research and the tone of positive convictions growing out of great experience. Not the least valuable feature of the paper is the clearness with which he has placed his conclusions before us. Remarks that might embrace all the points made by Dr. R., would not be desirable, so I will limit myself to such as I deem of most importance.

There is no doubt in my mind that the ovum may be fructified at any point during its transit, and form attachments to any part of the genital canal, from the distal extremity of the Fallopian tube to the cervical canal inclusive. Hence I should expect to find the placental implantation in cases of prævia at, and partially encroaching upon, the internal os, covering it centrally, and in the cervical cavity. When we remember that the placenta can attach itself to serous membrane—as in ventral pregnancy—the nature of the mucous membrane of the cervix cannot be adduced as an argument of sufficient cogency to prove this impossible. As to the source of the hæmorrhage, I think the opinion expressed by the writer the most plausible. The causes of the separation of the placenta are to be found in the difference in the structure and mode of development of the cervix as compared with the body and fundus. The muscular fibers are larger and more numerous in the fundus and fundal portion of the body, than in the lower zone of the body, and very much more so than in the neck. In normal pregnancy, the development or growth of the fundus is more rapid and more extensive than any part of the body, and the upper part of the corpus more and earlier than the lower, while the cervix is developed last of all. Not only is this true, but the character of development is different in the cervix as compared with the fundus and body. The enlargement of the fundus and body, especially the upper portion, is one of true hypertrophy. All the tissues entering into the structure of these portions of the uterus are increased sufficiently to accommodate that portion of the ovum occupying them without distension. The enlargement of the cervical portion of the organ, however, is partly hypertrophy and partly distension, and, I believe, in a less degree this is true of the lower or cervical portion of the body. The distension of the part of the body near the neck begins in the latter part of the eighth month, and that of the cervix is accomplished for the most part during the ninth month. At any rate, we can satisfy ourselves by examination that it progresses very rapidly during the last month. When gestation is finished, the cervical portion is large enough to contain the head, and its walls are comparatively thin. With this understanding of the structure and mode of development, I am inclined to consider the fundus and body as the contracting parts of the organ, while the cervix is the dilating portion. Probably the implantation of the placenta over, or in the cervix—by virtue of the greater supply of blood to the placental site of the organ—may to some extent modify the manner of development of the cervix, but it does not entirely do away with the manner of its enlargement. So soon, therefore, as the distension assumes its normal preponderance over the process of hypertrophy, the surfaces of attachment slide upon each other, a number of vessels are torn and hæmorrhage results. In central implantation this occurs early and becomes greater as the time for labor approaches. The treatment should begin at the time of the discovery of the condition, and be perseveringly continued until the cause is removed. It is the teaching of universal experience that a woman is in imminent danger every moment after hæmorrhage has begun. It is seldom that this symptom of placenta prævia shows itself before the fœtus is viable, and its safety depends upon a speedy release from its hazardous relation. Artificial premature delivery, according to the plan recommended and practiced by Greenhalgh, should be performed without hesitation, at the earliest practical moment after the diagnosis is clearly made out.

If our attention is not called to a case until labor has begun—and this will usually occur before term—energetic measures should be instituted, and the patient should not be left until delivery is completed, and the patient revived from the exhaustion usual in the most favorable cases. While I freely acknowledge that no one course of treatment will answer equally well in all cases, and that much must be left to the judgment of the immediate attendant, I believe we can settle upon a plan that will be generally applicable.

The principal means available at such times are the tampon to staunch the bleeding, ergot to promote the expulsion of the contents of the uterus, stimulants and nourishment to sustain the patient. If the hæmorrhage is not so urgent as to render it necessary to use the plug immediately, the evacuation of the liquor amnii will be of great service, not so much for the purpose of allowing the condensation of the uterine walls and construction of the vessels, as to stimulate the uterus to increased expulsive efforts. The evacuation of this fluid does, however, have an immediate effect in causing the presenting part to press upon the placenta and constricting the vessels by muscular contraction.

The tampon should be applied so as to perfectly prevent hæmorrhage. This can be done. I agree with Dr. Roler that cotton in pellets is the best material for the tampon. The pieces of cotton should be moistened with water and each one secured by twine, and the vagina packed so firmly that there is no room for the smallest stream of blood to escape. After the tampon is applied and the hæmorrhage is checked, it should be watched, and if necessary add to it and wait, not for an opportunity and sufficient dilatation to turn, but for the expulsion of the fœtus. While the tampon is being applied ergot ought to be given in full doses. By external examination the presentation can be ascertained, and corrected, and the uterus further stimulated and aided in its expulsive efforts by pressure with the hands upon the fundus and upper part of the body.

In some of these cases the after hæmorrhage is an item of great importance. When the implantation is central, or cervical, there is not sufficient contraction at the site of the attachment to entirely close the severed vessels; hence, although there may not be a large loss, there is a continued drain that may be enough to turn the scale against the already exsanguined patient. This may be materially lessened if not entirely checked by the direct application of astringents, or the hæmostatic preparations of iron. They may be applied by a large swab of cotton introduced up to the bleeding parts, and kept there until coagulation occurs, and then withdrawn. Of course the cautious practitioner will watch the uterus and promote its contraction by the usual means.

DR. MILLER said he fully agreed with the essayist regarding the cause of placenta prævia, viz., the ovum dropping in the cavity of the uterus till it is obstructed at the inferior segment, to which it becomes attached, and at that point the placenta is developed. The subject introduced this evening leads to the enquiry: What effect is produced in the uterus by pregnancy? Prominently the enlargement of the volume of the organ by increased nutrition. In normal pregnancy the development is manifested first in the fundus, and progresses downward till finally, in the last months of gestation, the cervix and os uteri are affected. Is the inferior portion enlarged by mechanical distension? He would associate with the theory of stretching, the idea of tension, then as a consequence, would not the tissue become firm and more resisting? We observe, however, that the tissue of the os and cervix becomes softer and more relaxed as term approaches. When the os uteri is felt hard and resisting, some pathological condition would explain it, in the supposed case.

In placenta prævia, the os and cervix are not thin, but much thicker than in normal pregnancy, in obedience to the law that the portion of the uterus to which the placenta is attached, is increased by more active nutrition, enlargement of vessels, sinuses, etc.

He inclines to the theory that the hæmorrhage in placenta prævia is due to vital action in the tissue of the uterus. The placenta is passive, the uterus is active. The placenta is developed by the end of the fourth month. No new elements enter into its composition after this period. The chorion villi have disappeared. After this period of gestation, the cervical zone of the uterus is developed; it is enlarged, by a process of growth, beyond the enlargement of the placenta; as a consequence, the attachment at and near the os uteri becomes so feeble that the weight and current of blood in the vessels and sinuses of the parts involved, rupture the feeble connection, and hæmorrhage follows. The hæmorrhage is "*unavoidable.*"

In regard to treatment, the suggestion made by Greenhalgh: that labor should be induced when the fœtus has attained viability, is worthy of consideration, and adoption. The dangerous hæmorrhage usually takes place after the seventh month. After this period, the uterus may be excited to action, while the attendant is present to observe the progress of the labor, and render assistance at the moment it may be required, and thus he may preserve the life of the child, and save the mother.

If hæmorrhage commences before the fœtus has become viable, it may be checked by a properly applied tampon, by position of patient, and by other means. But when desirable to excite uterine contractions during the hæmorrhage, the os uteri should be *strongly* compressed, and the upper part of the vagina *greatly* distended by the tampon. When the contractions of the uterus have been established, their efficiency may be increased by evacuating the liquor amnii.

The reason why cases sometimes terminate favorably, without assistance, when the placenta has been completely separated spontaneously, is doubtless because from the first the uterus contracts with energy.

Barnes would lead us to believe that safety in placenta prævia depends upon the *practical* separation of the placenta. Is this true? The essayist has answered the question in the negative; and this is what should be expected. If the irritation attending the partial separation should excite the uterus to efficient contractions, it is conceivable that the presenting part might be forced against the separated portion of the placenta, and this against the open vessels and sinuses of the uterus, and thus hæmorrhage be stayed, but unfortunately for the theory involved, the uterus does not always thus respond.

The source of the hæmorrhage he believed to be both from the separated portion of the placenta, and from the uterine vessels.

In answer to the question as to what material he used for a tampon, Dr. Miller said a strip of cotton cloth like a common roller, one end of which was introduced and the remainder carried up till the vagina was sufficiently filled. He had used sponges, but they were not always to be had at the time they were needed.

DR. CRAWFORD, of Monmouth, Ill., who was present by invitation, expressed his interest in the subject under discussion, and inquired how early in pregnancy a placenta prævia could make itself known. He narrated a case under his observation of a woman who suffered a violent hæmorrhage at the fifth month. By plugging the loss was arrested. The hæmorrhage recurred, however, at the sixth and seventh months, and during labor. The plugging was repeated each time, and the woman and child survived the labor. In answer to a question, Dr. Crawford said that his was a case of partial placenta prævia.

DR. DUDLEY: Previous to the year 1672, I believe, there is no record that any man recognized placenta prævia. Cases of dangerous flowing in puerperal women occurred, but no satisfactory reason was given. When the placenta was found at the os internum, it was supposed to have been detached from its normal position, and to have remained in its detached position.

In 1672 Paul Portal made investigations which resulted in the discovery of placenta prævia. I do not know that he suggested any very efficient means of treatment. His investigations passed out of mind and were lost. In the next century his discovery was independently re-discovered by several eminent obstetricians. They, in their turn, so far as I know, made no valuable suggestions of treatment. Forty years ago nothing was known of placenta prævia more than Portal knew in 1672.

Within forty years valuable additions to the means of treatment have been made, and among them is the tampon, introduced one hundred years ago, by Leroux; the water bag; puncture of the membranes, and detachment of the placenta. But even now, on the authority of Sir James Simpson, the mortality in placenta prævia is frightful. His statistics show that about one-third of all the mothers die, and about one-half of the children. We are, therefore, far from the position where we may rest and call the work done.

The practice of delivering prematurely, mentioned by Drs. Byford and Miller, was first described by Greenhalgh, of London, in 1866, but from the comments then made upon his pamphlet, it was evident that this practice was not uncommon among the leading obstetricians in Great Britain.

In 1868, Thomas, of New York, published several cases. Then supposing himself to be the pioneer in this treatment, and not knowing that Greenhalgh had anticipated him.

The reasons for premature delivery, *i. e.* delivery two months before term, are so obvious that it is remarkable that it was not thought of earlier, and much more remarkable that the practice is not more commonly employed. The question on the part of the mother is, shall we deliver at eight months before she has become exsanguined from loss of much blood, when we may be present during the entire progress of labor prepared to meet any emergency, or shall we wait until term, with the possible chance that death from sudden hæmorrhage may occur at any time and before aid can be called, and then deliver when the patient has become exhausted from repeated hæmorrhages and constant anxiety. But the question on the child's part is, does he have a better prospect at eight months out of the uterus, dependent upon pulmonary respiration, or shall he remain in the uterus, dependent upon placental aëration, which must necessarily be impaired proportionately to the amount of hæmorrhage, and then be subject to all the danger of perhaps a powerless labor in placenta prævia.

Dr. Thomas reports eleven cases treated by premature delivery, and of these only one mother was lost, and she died of puerperal septicæmia. Three of the children were still born; all of the remaining eight did well but one, who died in a few hours after delivery.

This gives Dr. Thomas in his eleven cases a percentage of about thirty-six deaths on the part of children, and a percentage of about nine on the part of mothers,—a vast improvement upon the statistics of Simpson. In all obstetric operations requiring a speculum, I am satisfied that Sims' is more applicable than any other, and should be more generally used. There is no doubt but that the tampon may be applied more efficiently through Sims' speculum than by any other means.

After introducing the speculum, pack as much cotton as possible around the cervix, distending the vaginal cul-de-sac to its uttermost, then partially withdraw the speculum and again introduce its blade in front of the cotton, and by making pressure backwards with the speculum you will have almost as much room for packing the cotton as before any was put in. The speculum may be thus withdrawn and re-introduced until the vagina is full. The amount of cotton possible to introduce in this manner, and therefore the amount of pressure about a bleeding uterus is really remarkable. Let me repeat that so valuable an instrument as Sims' speculum ought to have more general use in obstetrical operations.

DR. SAWYER: There are two points in connection with the subject before the society upon which I desire to speak.

First, I would ask if there is any evidence that an ovum once entirely or partially separated from its usual site near the fundus can form new attachments in the lowest zone?

This question is suggested by the history of a case under my observation, of a lady who, finding herself pregnant much against her wishes, made many efforts to rid herself of the ovum.

Among other measures, she described how she attempted to pass a male elastic catheter into her uterus, at least a dozen times during the first three months of pregnancy. Near her term I was first called to her on account of an alarming hæmorrhage, at midnight. I discovered a presentation of the placenta, and learned the history already narrated. She seemed anxious to confess to me, and demanded to know if her criminal attempts would explain the hæmorrhage, which was something that never happened in her previous pregnancies. A few weeks later, the unfortunate lady fell in labor and lost her life. An examination of the elliptical placenta revealed that its very center had been implanted over the internal os uteri. This fact was shown by an area of smooth fibrous-like tissue upon the uterine surface of the placenta, which apparently had formed no attachment with the uterine tissue.

The inquiry was raised: If this ovum was implanted over the internal os in the earliest days of pregnancy, would not the passage of the cathether have so disturbed its attachments that it would have been thrown off by the uterus? I know it will be offered that it is improbable that the catheter entered the uterus at all. But I am inclined to the belief that once, at least, the woman did traverse the ora, because the last effort, she told me, was followed by a profuse hæmorrhage, which so alarmed her that she was constrained to desist from further trials.

Is it unreasonable, I submit, that the ovum may have been dislodged from its usual site near the fundus by the instrument, say in three-fourths of its circumference, at an early period of gestation before the withering of the primitive villi, and have rolled down into the most dependent part of the uterine cavity, and there formed new attachments, while the remaining undetached villi were sufficient to insure the integrity of the ovum until the new attachments were completed?

Another point is one which I have never seen alluded to in the books, namely, the friability of the uterine tissue upon which the placenta is developed. In the case just referred to, the uterus was removed and examined immediately after death, and I was chagrined to find a tear which involved the entire thickness of the tissue proper of the uterus, but not the peritoneum, and about four inches in length—the entire length of the elongated neck,—upon the right side, at the junction of the anterior and posterior walls.

It was easy to mark out the placental site, and the ease with which this part was torn in my hands compared with the tearing of tissue at the fundus was something striking.

In the labor I used no undue violence. I experienced no difficulty in introducing my hand through the ora, nor in performing version. The chin of the large child passed over the right side of the neck, and may have caused the rupture. It is proper to add there was a constant dribbling of blood after delivery, though the post-partum contractions were complete; and this loss may have somewhat increased the shock which destroyed the patient.

This case recalled another which was under my care three years before. The woman survived the first shock from a labor with complete placenta prævia, but developed, within the first forty-eight hours, an attack of pelvic cellulitis, confined to the right half of the pelvis. Puerperal mania supervened, and death occurred on the fourteenth day after delivery. In this case version was performed, and the chin of the child (the latter weighing thirteen and three-quarters pounds) passed over the right side of the cervix. I am not able to say that the uterine tissue in the latter case was torn; in fact the speculum was never used; but the very rapid development of the cellulitis has led me to think that there was some lesion as its point of origin.

In the light of this experience I would, in future cases, use extreme care in all procedures which would stretch the uterine tissue in the cervical zone, upon which the placenta is attached.

A peculiar state of the cervix early in the labor in one of my cases of placenta prævia, is of sufficient interest, I think, to be spoken of in this connection. Its form was that of an inverted funnel. The external os was fully three centimetres in diameter; the internal os, on the contrary, barely admitted the end of the index finger, which impinged against the placental tissue. The length of the cavity of the cervix was as great as in the unimpregnated state. This state of things existed at term, when labor had actually begun; it shows the conservatism of nature in maintaining the spincter-function of the internal os, so that the placental attachment should not be disturbed until the external os was partly dilated. It also shows that the cavity of the cervix is not always merged into the cavity of the body of the uterus during the last days of pregnancy, as is usually held.

Finally, it has seemed to me that in point of grave significance the slight over-lapping of a flap of the placenta upon the cervical zone—and which is known by the ominous name of partial placenta prævia,—bears no comparison to a central implantation of the placenta.

Within a few weeks I have attended a labor, in which, during the very earliest moments of the first stage, there was an escape of blood, much more than what is known as the show, but not enough to occasion any alarm. The edge of the placenta was easily accessible upon the mother's left side. The pains were moderate, but soon the head fairly engaged, with the occiput posterior and to the woman's right side. The labor progressed and terminated favorably for both mother and child with absolutely no unusual interference.

DR. FITCH: I have but little to say upon this subject. I have enjoyed the paper and discussion, and feel better prepared to treat a case of placenta prævia than before.

My experience in this has been somewhat similar to that in puerperal inversion of the uterus. During my practice of 27 years, I have had but two or three cases, and none of them complete or central; not one of them requiring any special treatment.

Dr. John Bartlett read a paper on this subject, at the last regular meeting of the Chicago medical society. The plan of treatment suggested by him was to carry an instrument on the index finger, secured by a ring, into which the point of the finger passed; from this ring, and the point of the finger, extends a spear with serrated edges (if I understood him correctly), with which he pierces the placenta and then introduces through the opening thus made, a Hobbs' dilator, which, at the same time, acts as an efficient tampon and dilator. This device I think very ingenious, yet I am satisfied that the principal objection to this operation is that the complete separation of the placenta from the uterus, by hydrostatic pressure would almost necessarily follow, and the consequent death of the fœtus.

With regard to the source of the blood in this hæmorrhage, I believe that Dr. McKinzie has demonstrated the fact on the bitch that the principal part of the blood, if not all, comes from uterine sinuses.

DR. JONES called to mind the advice of the venerable Dr. Jonathan Knight, of Connecticut, who proposed that alum in powder or in lump, be used at the commencement of every tampon, whether the hemorrhages were "accidental" or "unavoidable." His own experience substantiated Dr. Knight's confidence in its hæmostatic value.

DR. NELSON: It has been my fortune to escape from most of these cases of placenta prævia. I have had but one case myself, though I saw Dr. Roler's case as reported, two days before delivery, and I have always felt very uncomfortable about that case, especially after I learned the unfortunate result of it, fearing I did not do my whole duty. I believe when such a case is discovered, so near term as this was, it should never be left without a medical attendant ready to act when necessary; for usually the greatest danger is the loss of blood occurring before the arrival of the physician. Probably no other result could have been obtained in this case if the woman had had skilled attention constantly from the time I saw her till Dr. Roler was finally called.

As to the cause of these unfortunate cases, I wish to emphasize the diseased condition of the mucous membrane of the uterus, such that when the fecundated ovum reaches the interior of the uterus, the mucous membrane does not respond as it should to retain the ovum in its depressions at the fundus, but from its unnatural smoothness, and the quantity and quality of the secretions, the ovum glides down to the inner os, and there forms its attachments, producing a case of placenta prævia or perhaps, fortunately escapes from the cervix and is entirely lost.

The only case of placenta prævia I have had myself, and that only partial, was in a patient I had treated for several months for chronic metritis. Dr. Roler's patient, I understand, had also been treated for chronic inflammation previous to this pregnancy. One, at least, of Dr. Sawyer's cases had had a severe pelvic cellulitis, resulting in abscess, and was subsequently treated for chronic metritis and perimetritis, during the most of a year previous to her last pregnancy.

It may be said, if chronic inflammation of the uterus is the cause of placenta prævia, this accident should be much more frequent. I reply that, fortunately, if the fecundated ovum does not become attached at the fundus of the uterus, it is usually passed from the cervix entirely, and the woman does not become pregnant. In all the cases I have seen, I am very sure the placenta was implanted within and over the internal os, not upon the walls of the cervical canal.

